# Plasma levels of acylated ghrelin in patients with insulinoma and expression of ghrelin and its receptor in insulinomas

**DOI:** 10.1007/s12020-020-02233-4

**Published:** 2020-03-02

**Authors:** Hai-Yan Wu, Nai-Shi Li, Yu-Li Song, Chun-Mei Bai, Qiang Wang, Yu-Pei Zhao, Yu Xiao, Shuang Yu, Ming Li, Yuan-Jia Chen

**Affiliations:** 1 Department of Gastroenterology, Peking Union Medical College Hospital, Chinese Academy of Medical Sciences, Peking Union Medical College, Beijing, 100730 China; 2 Department of Endocrinology, Peking Union Medical College Hospital, Chinese Academy of Medical Sciences, Peking Union Medical College, Beijing, 100730 China; 3 Department of Medical Oncology, Peking Union Medical College Hospital, Chinese Academy of Medical Sciences, Peking Union Medical College, Beijing, 100730 China; 4 Department of Surgery, Peking Union Medical College Hospital, Chinese Academy of Medical Sciences, Peking Union Medical College, Beijing, 100730 China; 5 Department of Pathology, Peking Union Medical College Hospital, Chinese Academy of Medical Sciences, Peking Union Medical College, Beijing, 100730 China

**Keywords:** Insulinoma, Ghrelin, Growth hormone secretagogue receptor (GHS-R), Insulin, Obesity, BMI

## Abstract

**Background:**

Insulinoma is a subtype of pancreatic neuroendocrine tumors. Many patients with insulinoma are obese due to frequent food intake. Ghrelin is associated with obesity and blood levels of insulin. It is not clear if plasma levels of ghrelin in insulinoma patients correlate with hyperinsulinemia and obesity. Expression of ghrelin and its receptor has not been well demonstrated in insulinoma.

**Objective:**

To study if plasma levels of ghrelin is associated with obesity and hyperinsulinemia or hyperproinsulinemia in patients with insulinoma, and to detect the expression of ghrelin and its receptor in insulinoma.

**Methods:**

Plasma levels of acylated ghrelin, insulin, and proinsulin were measured in 37 patients with insulinoma and 25 controls by ELISA. Expression of ghrelin and its receptor GHS-R1A was examined in 20 insulinoma and paired pancreatic specimens by immunostaining. *P* ≤ 0.05 was considered significant.

**Results:**

The plasma levels of acylated ghrelin in patients with insulinoma were significantly lower than that in the controls (median 15 pg/ml vs. 19 pg/ml, respectively, *P* = 0.016). The reduced plasma levels of acylated ghrelin in patients were significantly correlated with obesity, hyperinsulinemia, and hyperproinsulinemia (*P* = 0.029 and *P* = 0.028, respectively). Expression of ghrelin and its receptor GHS-R1A was shown in the majority of insulinoma specimens. The expression of GHS-R1A was positively correlated with ghrelin expression in insulinoma (*P* = 0.014).

**Conclusions:**

Plasma levels of acylated ghrelin decreased in patients with insulinoma, probably due to the hyperinsulinemia and obesity in the patients. Expression of both ghrelin and its receptor is common in insulinoma.

## Introduction

Insulinoma is one of the most common types of functional pancreatic neuroendocrine tumors (PNETs) [1–3]. Patients with insulinoma manifest the symptoms and signs of hypoglycemia due to hypersecretion of insulin/proinsulin by the tumor cells. Very few patients die due to malignant tumor (with metastasis). However, due to hunger or for preventing hypoglycemia, hyperphagia leads many patients with insulinoma to weight gain or obesity [4, 5], which then impairs patients’ quality of life. The gut hormone ghrelin is mainly secreted by the stomach [6] and exerts various biological/physiological functions, including effects on secretion of other hormones, glucose homeostasis, pancreatic function, cell proliferation, memory, gastric acid secretion, immunity, energy expenditure, the hypothalamic–pituitary–adrenal axis, and others [7, 8]. Interestingly, it has been reported that ghrelin is an orexigenic peptide and closely associated with obesity by increasing body weight in humans [9]. Endogenous ghrelin stimulates hunger and food intake more effectively than any other known peptides except neuropeptide Y, with which it is approximately equipotent; and exogenous injection of ghrelin also stimulates food intake rapidly by increasing appetite and the number of meals [10–13]. Moreover, ghrelin has been reported to stimulate appetite and food intake even more in obese than lean humans [14]. Most biological actions of ghrelin, especially those involving endocrine and anabolic effects, require acylation [7, 10, 15, 16]. Acylation of ghrelin is also required for activating its functional receptor in vivo [6]. It was reported that insulin could reduce blood levels of ghrelin independently of glucose levels in healthy control [17]. We wonder whether peripheral blood levels of acylated ghrelin in patients with insulinoma are associated with their obesity. In addition, active uncontrolled oversecretion of insulin by insulinoma is not regulated by the feedback of blood glucose, and this is the primary feature of insulinoma and the basis of hypoglycemia. However, it is not clear whether blood levels of acylated ghrelin are affected by higher circulating levels of insulin/proinsulin in patients with insulinoma. On the other hand, ghrelin inhibits the secretion of insulin by rodent insulinoma cell lines, and administration of ghrelin to the human pancreas inhibited arginine-stimulated insulin secretion [18, 19].

Growth hormone secretagogue receptor type1A (GHS-R1A) is the functional receptor of ghrelin [10, 20, 21], which is expressed widely in human body (including a number of endocrine organs such as the pituitary, pancreas, and thyroid). It has been shown that ghrelin and GHS-R1A are expressed in neuroendocrine tumors [22–29]. However, whether ghrelin and its functional receptor are expressed in insulinoma tissues and whether the expression of ghrelin and GHS-R1A correlate with plasma level of ghrelin in patients with insulinoma have not been well elucidated.

Therefore, in present study, we focused on the blood levels of acylated ghrelin, insulin, and proinsulin simultaneously in patients with insulinoma as well as the expression of ghrelin and its receptor GHS-R1A in insulinoma tissues. We want to address whether the plasma levels of acylated ghrelin is correlated with obesity, the plasma levels of insulin or proinsulin, and the expression of ghrelin and its receptor in patients with insulinoma. Furthermore, the blood levels of acylated ghrelin in patients and the expression of ghrelin and its receptor in tumor were correlated with clinicopathological features of the patients.

## Material and methods

### Patients, controls, samples collection, and clinicopathological features

The study was conducted at Peking Union Medical College Hospital as a single center enrolled 37 Chinese patients with insulinomas and 25 volunteer participants as controls from 2004 to 2018. The controls were matched with the patients on age, gender, and BMI. The volunteer controls were excluded if anyone had endocrine disorders such as diabetes, endocrine tumors, metabolic syndrome, past gastric surgery, kidney or liver function abnormalities, using glucocorticoids or eating disorders. The volunteer controls were given their written informed consent before inclusion into the study.

The diagnostic criteria for insulinoma were reported previously [30–32]. Briefly, the clinical and laboratory diagnostic criteria of insulinoma included symptoms of hypoglycemia, serum levels of glucose <50 mg/dl, and elevated serum levels of insulin or proinsulin at time of hypoglycemia. The tumors were mainly localized by computed tomography with contrast, magnetic resonance imaging, and endoscopic ultrasound. The pathological diagnosis was made by an experienced pathologist. We analyzed tumor grade and stage of these patients according to ENETS guideline.

Preoperative blood samples from 37 fasted patients with insulinoma and blood samples from 25 fasted volunteer controls were obtained. Plasma was isolated from blood and stored at −80 °C until assay.

### Chinese criteria of obesity

According to the Chinese criteria of weight for adults (issued by National Health Commission of the People’s Republic of China in 2013, see Supplementary file #1), criteria of obesity in Chinese is BMI > 28. All of the patients and controls in the present study are Chinese, thus, we defined that an individual with BMI ≥ 29 is obese.

### Isolation and purification of plasma acylated ghrelin and assays of acylated ghrelin, insulin, and proinsulin

Most biological function of ghrelin require acylation. The acylated ghrelin was extracted and purified according to the manufacturer’s protocol, the plasma levels of acylated ghrelin were measured according to the protocol (Bachem, Switzerland). Briefly, 1 ml of plasma was loaded onto a Sep-Pak C18 cartridge (Waters, USA) pre-equilibrated with 5 ml methanol and 5 ml deionized water. The cartridge was washed with 5 ml deionized water and eluted with 2 ml 90% methanol. The elution was evaporated, lyophilized, and dissolved in the buffer provided by the Enzyme Immunoassay (EIA) kit (S-1222, Bachem, Switzerland). The EIA was carried out by using the competitive method with antibody against acylated ghrelin as the protocol described. All samples were measured in duplicate.

Plasma levels of insulin and proinsulin were concurrently measured by using ELISA methodology as we reported previously [33, 34].

### Detecting of ghrelin and its receptor GHS-R1A expression in insulinomas

Among the 37 patients whose plasma levels of acylated ghrelin were detected, we collected 20 tumor specimens due to tissue availability. Expression of ghrelin and its functional receptor GHS-R1A was detected in 20 formalin-fixed and paraffin-embedded specimens of insulinoma by immunohistochemical staining (IHC), and in 20 paired para-tumoral tissue specimens. After antigen retrieval (microwave heating, 4 min in 0.01 M citrate sodium, pH 6.7, for 3 times), sections were incubated with rabbit antibody against ghrelin at 1:800 dilution (Phoenix Pharmaceuticals, Inc., H-031-30) for 2 h at room temperature, and parallel sections of the specimens were stained with rabbit antibody against human GHS-R1A at 1:600 dilution (Phoenix Pharmaceuticals, Inc., H-001-62) for 1.5 h, at room temperature. Donkey anti-rabbit IgG/horseradish peroxidase (Beijing Zhongshan Golden Bridge Biotechnology Co.) was used as second antibody, and diaminobenzidine used as chromogen. The results were interpreted by two persons blinded to circulating levels of ghrelin and clinical data. The criteria of semiquantitative grading of IHC were similar to our previous reports [30, 35].

### Statistical analysis

SPSS statistics software version 20.0 was used for statistical analysis. Significance was calculated using the Fisher’s exact test, *χ*^2^-test, Mann–Whitney *U* test, Kruskal–Wallis *H* test and Student *t* test. Two-tailed test was used in all of statistical analysis. *P* ≤ 0.05 was considered significant.

## Results

### Clinicopathological characteristics

We studied 37 patients with insulinoma. All the insulinomas were well-differentiated and most of them (82%) were G1 and others were G2. According to the ENETS guideline, stage was assessed in the 37 patients. Of the 22 patients followed, 20 patients (91%) were alive without disease while 2 patients were alive with liver metastasis. The clinicopathological features of 37 patients were summarized in Table 1.

**Table 1:** Summary of clinicopathological features of insulinoma patients

Clinical features	*n* = 37
Gender, *n* (%)	
Male	17 (45.9)
Female	20 (54.1)
Median age at diagnosis, *y* (range)	44 (18–74)
Median body mass index (BMI) (range)	27.6 (19.3–39.6)
BMI ≥ 29, *n* (%)	13 (35.1)
BMI < 29, *n* (%)	24 (64.9)
Primary tumor location, *n* (%)	
Pancreatic head and/or neck	19 (51.4)
Pancreatic body and/or tail	15 (40.5)
Both of pancreatic head and body	2 (5.4)
Tumor size, *n* (%)	
<2 cm	22 (62.9)
≥2 cm	13 (37.1)
Metastasis, *n* (%)	
No metastasis	35 (94.6)
Metastasis	2 (5.4)
Ki-67, *n* (%)	
≤2%	28 (82.4)
>2%	6 (17.6)
Grade, *n* (%)	
1	28 (82.4)
2	6 (17.6)
Stage, *n* (%)	
I	20 (57.1)
II	13 (37.1)
III	0
IV	2 (5.7)
Follow-up information	
Available	22 (59.5)
Not available	15 (40.5)
Follow-up months, median (range)	60 (12–267)
Disease-free survival (DFS), *n* (%)	20 (90.9)
Died of disease (DOD), *n* (%)	2 (9.1)

Grade and Ki-67 data were from 34 patients, tumor size and stage data were from 35 patients

The controls were matched with the patients on age, gender, and BMI. About 35% of patients were obese (BMI ≥ 29), similarly 32% of controls were obese. The median age of both patients and controls were the same (44 years old), 54% of patients were female, and 56% of controls were female. There was no significance of BMI, age, and gender between the patients and the controls.

### Plasma levels of acylated ghrelin, insulin, and proinsulin in patients and controls

The plasma levels of insulin and proinsulin in the patients with insulinoma were significantly higher than those in control group (*P* = 1.27 × 10^−6^ and *P* = 4.19 × 10^−9^, respectively), (Table 2). The normal cutoff value of insulin and proinsulin was ≤11 mU/L and ≤5 pmol/L, respectively [33, 34]. Neither plasma levels of insulin nor proinsulin was elevated in ten patients (10/37, 27%).

**Table 2:** Hormone levels in patients with insulinoma and control group

Hormone levels	Insulinoma *n* = 37	Control *n* = 25	*P* value
Ghrelin (median, pg/ml)	15	19	0.016
Insulin (mU/L)	29.8 ± 10.0	4.0 ± 0.7	1.27 × 10^−6^
Proinsulin (pmol/L)	24.8 ± 5.0	2.1 ± 0.3	4.19 × 10^−9^

Plasma levels of ghrelin in patient were significantly lower than that in the control group. Plasma levels of insulin and proinsulin were significantly higher than that in the control group, respectively

**Fig. 1:**
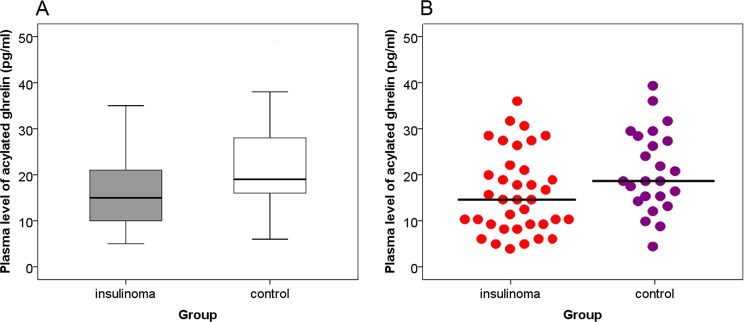
**a** Plasma levels of acylated ghrelin in patients with insulinoma and in control group. Acylated ghrelin levels in patients with insulinoma were significantly lower than that in the control groups [median 15 (5–35) vs.19 (6–47), *P* = 0.016], (mean ± SD 16.3 ± 8.2 vs. 22.0 ± 9.5). **b** Each dot represents an individual level of ghrelin in each patient

The plasma levels of acylated ghrelin in patients with insulinoma were significantly decreased than that in the control group (insulinoma median 15 pg/ml, range 5.0–35.0 pg/ml vs. control median 19.0 pg/ml, range 7.0–47.0 pg/ml, *P* = 0.016), see Table 2 and Fig. 1a, b. In the experiments, the coefficient of variation was 5.1%.

**Fig. 2:**
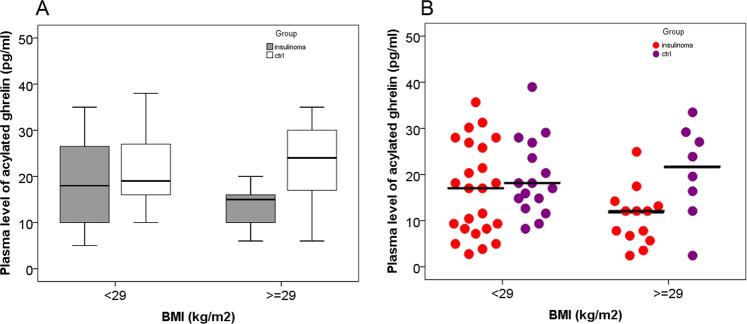
**a** Obesity could influence the plasma levels of acylated ghrelin in patients with insulinoma and control group. Plasma levels of acylated ghrelin in obese patients (BMI ≥ 29 kg/m^2^) were significantly lower than that in the obese controls [median 15 (6–27) pg/ml vs. 24 (6–35) pg/ml, *P* = 0.029], (mean ± SD 15.9 ± 7.7 vs. 21.1 ± 9.7). **b** Each dot represents an individual level of ghrelin in each patient

To investigate the reason that might reduce the plasma levels of acylated ghrelin in patients with insulinoma, we compared the plasma levels of acylated ghrelin in obese patients with those in obese volunteer controls, and the levels of acylated ghrelin in nonobese patients (BMI < 29) with that in nonobese controls. We found that the plasma levels of acylated ghrelin in obese patients were significantly lower than those in the obese controls (median 15.0 pg/ml vs. 24.0 pg/ml, *P* = 0.029) (Fig. 2a, b, right column). In contrast, the plasma levels of acylated ghrelin in nonobese patients were not different from those in the nonobese controls, *P* > 0.05 (Fig. 2a, b, left column). Thus, the finding suggested that reduced plasma levels of acylated ghrelin in patients with insulinomas could be, at least partly, due to the obesity of the patients.

**Fig. 3:**
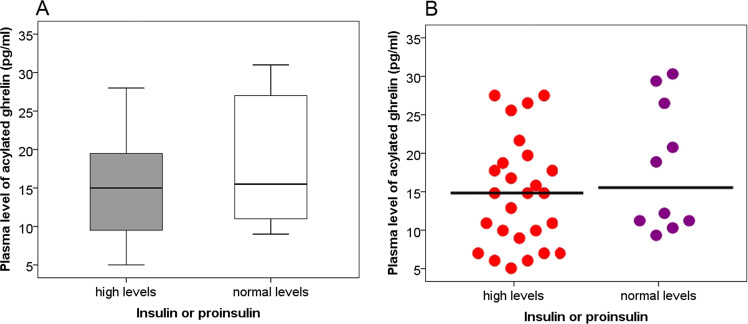
**a** Plasma levels of acylated ghrelin in patients with higher levels of insulin/proinsulin were significantly lower than that in patients with normal levels of insulin/proinsulin [median 15 (5–35) pg/ml vs. 19 (6–47) pg/ml, *P* = 0.028]. **b** Each dot represents an individual level of ghrelin in each patient

Hyperinsulinmia was reported to inhibit the secretion of ghrelin [17]. High levels of insulin and proinsulin are common in the patients with insulinomas. Thus, we aimed to study whether hyperinsulinemia or hyperproinsulinemia could impact the circulating levels of acylated ghrelin in patients with insulinoma. Compared with the patients with normal plasma levels of insulin or proinsulin (*n* = 10), significantly reduced plasma levels of acylated ghrelin can be seen in patients with elevated levels of insulin and proinsulin (*n* = 27, *P* = 0.028), Fig. 3a, b.

### Expression of ghrelin and its functional receptor GHS-R1A in insulinomas and controls

**Fig. 4:**
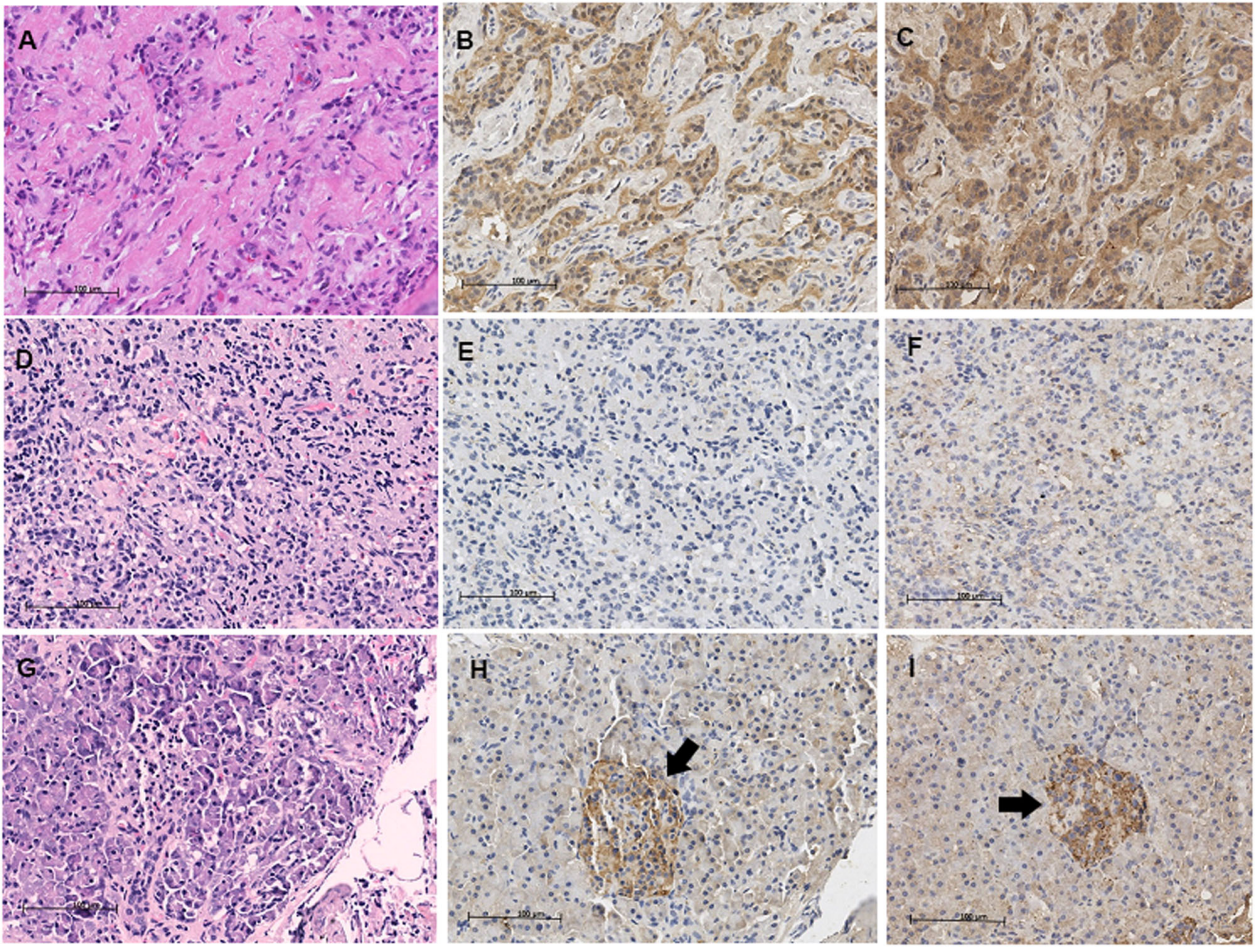
HE and immunohistochemical staining of ghrelin and its functional receptor GHS-R1A in insulinoma tissues and paired para-tumoral tissues. Left panel: HE staining; middle panel: ghrelin IHC, 10×; right panel: GHS-R1A IHC, 10×. scale bar, 100 μm. HE staining on an insulinoma tissue (**a**); positive IHC staining of ghrelin and its receptor GHS-R1A (**b** and **c**, respectively), in the same tumor tissue, shown the negative immunostaining of ghrelin and GHS-R1A in interstitial tissues (**b** and **c**); HE staining on another insulinoma (**d**) and its paired para-tumoral tissues (**g**); shown the negative IHC staining of ghrelin and its receptor GHS-R1A (**e** and **f**, respectively), in the same tumor tissue, while positive expression of ghrelin and its receptor GHS-R1A can be seen in islets within its paired para-tumoral tissue (**h** and **i**, respectively). Black arrows indicating pancreatic islets (**g**, **h**, and **i**)

Positive expression of ghrelin was shown in 16 of 20 (80%) insulinomas and in all of 20 paired pancreatic specimens adjacent to the tumors (20/20, 100%); the difference in expression rate did not reach significance (*P* = 0.069). In para-tumoral pancreatic specimens, immunostaining of ghrelin was found in the islets in all of the 20 specimens. HE and IHC staining of ghrelin in insulinoma and para-tumoral tissues as control were shown in Fig. 4.

Positive expression of ghrelin functional receptor GHS-R1A was found in 12 of 20 (60%) insulinomas and in 18 of 20 (90%) paired para-tumoral specimens (Fig. 4); the difference did not reach significance (*P* = 0.059).

Of the 12 insulinomas with positive GHS-R1A expression, all had expression of ghrelin (12/12, 100%), whereas expression of ghrelin was only found in 4 of 8 insulinomas without GHS-R1A expression (4/8, 50%), *P* = 0.014, see Table 3. The positive correlation between ghrelin expression and its receptor expression in insulinoma suggests that ghrelin might act on the tumor through autocrine or paracrine pathway.

**Table 3:** Relationships between expression of ghrelin and its functional receptor GHS-R1A in insulinomas

	Ghrelin + (*n*)	Ghrelin − (*n*)	*P* value
GHS-R 1 A+	12	0	
GHS-R 1A−	4	4	0.014

The expression of GHS-R1A in insulinoma tissues was positively correlated with its ligand expression

**Fig. 5:**
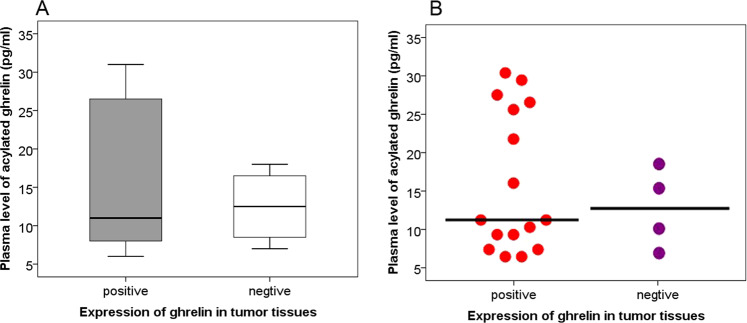
**a** Plasma levels of acylated ghrelin in patients with expression of ghrelin in insulinomas were not significantly different from that in patients without expression of ghrelin in tumor tissues [median 11 (6–31) pg/ml vs. 12.5 (7–18) pg/ml, *P* = 0.740]. **b**Each dot represents an individual level of ghrelin in each patient

We found that the plasma levels of acylated ghrelin in patients whose tumor expressing ghrelin was not significantly different from those in patients whose tumor not expressing ghrelin, *P* = 0.740, see Fig. 5a, b.

### Correlation of plasma levels of acylated ghrelin and expression of ghrelin and its receptor with clinicopathological characteristics

The plasma levels of acylated ghrelin in patients with insulinomas were not associated with clinicopathological features (Table 4).

**Table 4:** Plasma levels of acylated ghrelin in patients with insulinoma and clinicopathological characteristics

Clinical characteristics	*n*	Ghrelin levels (pg/ml)	*P* value
Age	37		
Gender			
Male	17	15 (7–35)	0.562
Female	20	15 (5–28)	
Primary tumor location			
Pancreatic head and/or neck	19	17 (9–31)	0.061
Pancreatic body and/or tail	15	10 (5–35)	
Tumor size			
≥2 cm	13	11 (6–35)	0.402
<2 cm	22	15 (5–31)	
Metastasis			
No metastasis	35	15 (5–35)	0.737
Metastasis	2	16 (12–20)	
Grade			
1	28	15.5 (5–35)	0.108
2	6	10 (6–20)	
Stage, *n* (%)			
I	20	16.5 (5–31)	0.803
II	13	11 (6–35)	
IV	2	16 (12–20)	

The expression of ghrelin in the tumor tissues did not correlate with any clinicopathological characteristics (Table 5). The expression of GHS-R1A in the tumor did not correlate with the clinicopathological features, except for the age; the patients with expression of GHS-R1A in tumor were much older than those without GHS-R1A expression in tumor (*P* = 0.041) (Table 5).

**Table 5:** Correlation of expression of ghrelin and receptor GHS-R1A with clinicopathological features

Clinical characteristics	*n*	Ghrelin (+)	Ghrelin (−)	*P* value	GHS-R1A (+)	GHS-R1A (−)	*P* value
Gender, *n* (%)							
Male	9	7 (78)	2 (22)	1.000	5 (56)	4 (44)	1.000
Female	11	9 (82)	2 (18)		7 (64)	4 (36)	
Age (years, *X* ± *S*_*X*_)	20	47.3 ± 15.7	38.3 ± 12.1	0.301	51.0 ± 16.3	37.1 ± 8.6	0.041
BMI (kg/m^2^, *X* ± *S*_*X*_)	20	28.5 ± 5.4	25.8 ± 3.8	0.365	29.2 ± 5.8	26.2 ± 3.7	0.214
Tumor location, *n* (%)							
Pancreatic head or neck	10	8 (80)	2 (20)	1.000	7 (70)	3 (30)	1.000
Pancreatic body or tail	9	7 (78)	2 (22)		5 (56)	4 (44)	
Tumor size, *n* (%)							
≥2 cm	9	9 (100)	0	0.094	6 (67)	3 (33)	0.670
<2 cm	11	7 (64)	4 (36)		6 (55)	5 (46)	
Grade, *n* (%)							
1	16	13 (81)	3 (19)	1.000	9 (56)	7 (44)	0.619
2	4	3 (75)	1 (25)		3 (75)	1 (25)	
Stage, *n* (%)							
I	11	7 (64)	4 (36)	0.094	6 (55)	5 (46)	0.670
II	9	9 (100)	0 (0)		6 (67)	3 (33)	

## Discussion

There are several reasons for studying the alteration of circulating levels of ghrelin in insulinoma. First, compared with other subtypes of PNETs, many patients with insulinoma are obese. Thus, it is reasonable to investigate whether ghrelin, which can increase body weight in humans, is associated with the obesity of patients with insulinoma. However, due to the limited cases of insulinoma, most of previous studies on blood levels of ghrelin in NETs or PNETs did not focus on insulinomas. Second, most recently, a clinical trial shows that, in 135 patients with functional neuroendocrine tumors, body weight loss is associated with uncontrolled carcinoid syndrome and reduced survival [36]. The finding suggested that body weight might be correlated with prognosis of neuroendocrine tumors. Third, it was reported that exogenous administration of insulin could suppress circulating ghrelin independently of glucose levels in healthy volunteers [17]. We think that insulinoma, as a “natural” model of endogenous oversecretion of insulin, could be used to evaluate the effect of insulin and proinsulin on circulating levels of ghrelin in the patients. On the other hand, the effect of ghrelin on insulin secretion is controversial, either stimulating the secretion of insulin [37] or inhibiting the secretion of insulin [18, 38, 39]. Therefore, we try to understand the potential interaction between the insulin and ghrelin, both of which are associated with obesity [9, 10, 40]. Finally, the acylated ghrelin is the primary form to exert its biological activity [6, 7, 41]. The majority of the previous studies on neuroendocrine tumors have not examined circulating levels of acylated ghrelin [27, 42, 43]. These researchers suggested that it was worthwhile to test acylated ghrelin levels in patients with NETs [41, 42]. A recent study on NETs compared fasting acylated ghrelin and unacylated ghrelin levels between NET patients and controls, showed that there was no significant difference between patients and controls in fasting acylated ghrelin but only three patients with PNETs were included and, obese patients and obese matched controls were excluded in this study [44].

We found plasma levels of acylated ghrelin in patients with insulinoma were significantly lower than those in the controls. Our results are different from Corbetta’s finding showing plasma ghrelin levels in patients with GEP tumors were similar to those in the controls [22]. However, only two patients with insulinomas were included in that study [22]. Another study showed that elevated serum levels of ghrelin in 85% of patients with hepatic metastatic NETs when compared with the standard reference range given for matched healthy controls [42]. Those previous studies on NETs measured circulating levels of total ghrelin but not acylated ghrelin. Another reason for different results between the studies on ghrelin levels is that different combination of NETs enrolled in different studies. Both Wang et al. and Corbetta et al. [22, 42]. studied not only pancreatic but also gastrointestinal NETs. Our research, however, focuses on insulinoma only, and obese patients, as well as obese controls are included. To the best of our knowledge, there is little work done on detecting circulating levels of acylated ghrelin in patients with insulinoma.

As mentioned above, insulin may reduce circulating levels of ghrelin independently of glucose in healthy control [17]. Our result also showed that plasma levels of acylated ghrelin were reduced in patients with high levels of insulin/proinsulin comparing with those in patients without high levels of insulin/proinsulin, suggesting high levels of insulin/proinsulin might inhibit plasma levels of acylated ghrelin in patients. Obesity might lead to declined plasma levels of acylated ghrelin in patients with insulinoma probably due to the negative feedback. One study reported a positive correlation between BMI and serum ghrelin levels in patients with NETs [42]. The difference could be due to the different tumor types between our study (insulinoma only) and theirs [42]. The plasma levels of acylated ghrelin did not correlate with clinicopathological characteristics which is consistent with previous findings [44].

In our present study, the plasma levels of acylated ghrelin are much lower than plasma levels of total ghrelin shown in other studies. One reason is that acylated ghrelin constitutes only a small part of total ghrelin; normally acylated ghrelin accounts for <10% of the total ghrelin in the circulation [8, 10]. Another reason might be that a small part of ghrelin is lost when we extracted and purified plasma ghrelin by using Sep-Pak C18 cartridge according to the manufacturer’s protocol.

The present study showed that positive expression of ghrelin was seen in 80% of insulinoma tissues, which is similar to the previous results on PNETs [27] and gastric carcinoid [23]. Positive immunostaining of ghrelin was also seen in islets and acini of peri-tumoral pancreatic tissues, similar to what was described in previous studies [45, 46]. Consistent with the result of one earlier study [27], the plasma levels of acylated ghrelin were not associated with the expression of ghrelin in tumor tissues in our study, suggesting majority of circulating ghrelin in patients mainly secreted by the stomach and small intestine as usual rather than from the tumor cells. Another reason is that ghrelin was synthesized in insulinoma cells but might not be secreted into circulation. Similar phenomenon was seen in our previous study showing the strong expression of chromogranin A in insulinoma cells while serum levels of chromogranin A in patients were not elevated [32].

GHS-R1A was the functional receptor of ghrelin [21, 47]. We found 60% of insulinomas expressed GHS-R1A, similar to the results of Ekeblad’s study [27] and Volante’s study [25]. The expression of ghrelin and its functional receptor was also seen in a number of hormone-related tumors, such as breast cancer, prostate cancer [48], pituitary adenoma, thyroid tumors, ovarian tumors [49], and a bronchial ectopic ACTH-secreting carcinoid [26]. The expression of GHS-R1A in insulinom tissues was not associated with plasma levels of acylated ghrelin.

Interestingly, we observed that the expression of GHS-R1A in insulinomas was positively correlated with its ligand (ghrelin) expression (*P* = 0.014). All of the 12 insulinomas which expressed receptor had the positive expression of ghrelin (100%), suggesting that ghrelin may act on the insulinoma through autocrine or paracrine pathway. A previous study showed that ghrelin exerted many biological functions, including effects on cell proliferation. It is interesting to investigate whether acylated ghrelin can regulate the growth of insulinoma cells in the future.

One of the limitations in present study is the relatively small number of patients due to the relatively rare tumors. Another limitation is that it would be better to detect both acylated ghrelin (active form ghrelin) and total ghrelin, but it needs more volume of blood (plasma), unfortunately, we did not collect more blood from these patients.

In conclusion, our findings suggested that plasma levels of acylated ghrelin were decreased in patients with insulinoma, probably due to the obesity and endogenous hyperinsulinemia in the patients. Expression of both ghrelin and its functional receptor is common in insulinomas.

## Supplementary information

Supplementary Information
