# Supplementary material for: Plasma levels of acylated ghrelin in patients with insulinoma and expression of ghrelin and its receptor in insulinomas
Source: Endocrine. 2020 Mar 2;68(2):448–57. doi: 10.1007/s12020-020-02233-4 (PMC7266859; doi:10.1007/s12020-020-02233-4)
Supplement: Supplementary file 1 — Supplementary Information [file 12020_2020_2233_MOESM1_ESM.pdf]

ICS 11.020
C 55

WS

中华人民共和国卫生行业标准

WS/T 428—2013

成人体重判定

Criteria of weight for adults

2013-04-18 发布

2013-10-01 实施

中华人民共和国国家卫生和计划生育委员会 发布

前　　言

本标准按照 GB/T 1.1—2009 给出的规则起草。

本标准起草单位：新探健康发展研究中心、中国疾病预防控制中心营养与食品安全所、中国疾病预防控制中心、北京大学公共卫生学院、中国医学科学院阜外心血管病医院、首都医科大学附属安贞医院、国际生命科学学会中国办事处、上海交通大学附属第六人民医院。

本标准主要起草人：陈春明、赵文华、杨晓光、陈君石、李可基、赵连成、姚崇华、赵熙和、李光伟、贾伟平、顾东风、赵冬、杨正雄。

成人体重判定

1 范围

本标准规定了成人体重的判定、测量条件和测定方法。

本标准适用于成人(18 岁及以上)超重和肥胖及中心型肥胖的判定,可用于流行病学筛查和临床初步诊断,但不适用于某些特殊人群,如运动员、孕产妇等。

2 规范性引用文件

下列文件对于本文件的应用是必不可少的。凡是注日期的引用文件,仅注日期的版本适用于本文件。凡是不注日期的引用文件,其最新版本(包括所有的修改单)适用于本文件。

WS/T 424 人群健康监测人体测量方法

3 术语和定义

下列术语和定义适用于本文件。

3.1

身高 height

站立位足底到头部最高点的垂直距离。

3.2

体重 weight

人体的总重量。

3.3

体重指数 body weight index;BMI

体质指数

一种计算身高别体重的指数,计算方法是体重(kg)与身高(m)的平方的比值。

3.4

腰围 waist circumference

腋中线肋弓下缘和髂嵴连线中点的水平位置处体围的周径长度。

3.5

超重和肥胖 overweight and obesity

由于体内脂肪的体积和(或)脂肪细胞数量的增加导致的体重增加,或体脂占体重的百分比异常增高,并在某些局部过多沉积脂肪,通常用 BMI 进行判定;脂肪在腹部蓄积过多称为中心型肥胖(central obesity),通常用腰围进行判定。

4 体重判定

4.1 体重分类

以 BMI 为依据对成人体重分类,见表 1。

表 1 成人体重分类

分类	BMI 值<br>kg/m²
肥胖	BMI≥28.0
超重	24.0≤BMI<28.0
体重正常	18.5≤BMI<24.0
体重过低	BMI<18.5

4.2 中心型肥胖

中心型肥胖可以腰围直接判定，见表 2。

表 2 成人中心型肥胖分类

分类	腰围值<br>cm
中心型肥胖前期	85≤男性腰围<90<br>80≤女性腰围<85
中心型肥胖	男性腰围≥90<br>女性腰围≥85

5 测量条件和测量方法

参照 WS/T 424。
